# Comparison of a Fully Synthetic Electrospun Matrix to a Bi-Layered Xenograft in Healing Full Thickness Cutaneous Wounds in a Porcine Model

**DOI:** 10.7759/cureus.1614

**Published:** 2017-08-27

**Authors:** Matthew R MacEwan, Sarah MacEwan, Anna P Wright, Tamas R Kovacs, Joel Batts, Luke Zhang

**Affiliations:** 1 Research & Development, Acera Surgical, Inc; 2 Research, Telos Partners, Llc; 3 Research, Sinclair Research Center

**Keywords:** animal, healing, histopathology, synthetic, wound, matrix

## Abstract

A fully synthetic electrospun matrix was compared to a bi-layered xenograft in the healing of full thickness cutaneous wounds in Yucatan miniature swine. Full thickness wounds were created along the dorsum, to which these matrices were applied. The wound area was measured over the course of healing and wound tissue was scored for evidence of inflammation and healing. Animals were sacrificed at Day 15 and Day 30 and tissue samples from the wound site were harvested for histopathological analysis to evaluate inflammation and tissue healing as evidenced by granulation tissue, collagen maturation, vascularization, and epithelialization. Average wound area was significantly smaller for treatment group wounds compared to control group wounds at 15 and 30 days ([7.7 cm^2 ^± 0.9]/[3.8 cm^2 ^± 0.8]) and ([2.9 cm^2 ^± 1.1]/[0.2 cm^2 ^± 0.0]) (control/treatment) (p = 0.002/p = 0.01). Histopathological analysis of wound sections revealed superior quality of healing with treatment group wounds, as measured by inflammatory response, granulation tissue, and re-epithelialization. A fully synthetic electrospun matrix was associated with faster rates of wound closure characterized by granulation tissue, deposition of mature collagen and vascularization at earlier time points than in wounds treated with a bi-layered xenograft. Treatment with this fully synthetic material may represent a new standard of care by facilitating full-thickness wound closure while eliminating the risks of inflammatory response and disease transmission associated with biologic modalities.

## Introduction

The process of wound healing requires a coordinated effort of cellular recruitment and tissue growth. Regenerative matrix materials have been used to promote this coordination and provide immediate wound coverage to minimize the risks associated with infection and fluid loss. An ideal material for these purposes would serve as a healing scaffold, limit infection risk, minimize inflammation, be readily available for use, and be conformable to diverse wound surfaces [1-2].

Human autografts and allografts, animal-based xenografts, and fully synthetic materials have been used clinically with varying degrees of success. Autograft availability is limited by definition, and creates iatrogenic morbidity at donor sites. Allografts and xenografts eliminate the morbidity associated with autografts, but introduce additional risks of inflammatory response and disease transmission. Furthermore, host rejection of allografts and xenografts remains a concern in wound populations where the rate of immune disease has been reported as high as 23% [3].

To support the infiltration of fibroblasts, the deposition of new collagen, and re-epithelialization of the wound surface, the wound matrix material should persist in the wound for at least three weeks [4]. The timing of wound matrix degradation is therefore critical, as the wound matrix should resist degradation until sufficient new tissue growth has occurred. The susceptibility of biologic wound matrix materials to enzymatic degradation leads to a resorption rate that is poorly controlled, risking the premature degradation of the wound matrix prior to sufficient wound healing [5].

There remains a need for materials that minimize inflammation and promote complete wound healing [6-8]. Electrospun fibrous scaffolds may meet this need by mimicking the structure of native extracellular environment while enabling resident cells to perform their roles in the healing cascade [9-10]. The purpose of the following study was to compare a novel fully synthetic material to a commercially available bi-layered xenograft material using a porcine full-thickness wound model.

## Materials and methods

This study was approved by the Institutional Animal Care and Use Committee (Sinclair Research Center, Animal Care and Use Committee; Protocol #: D14119) and closely monitored by veterinarians to ensure that proper care and handling of the animals were provided.

Surgical procedure

A swine model was selected due to its applicability in translating results for potential clinical uses. Two Yucatan miniature swine were fasted overnight then clipped to remove hair from the dorsal-lateral area, which were then prepared for surgery with disinfectant and isopropyl alcohol. These preparations were performed by a single investigator. Anesthesia was administered with intramuscular injections of Telazol (~2.2 mg/kg) and Xylazine (~0.44 mg/kg) followed by Isoflurane for the duration of the surgical procedure (0.5-5.0% in 100% oxygen). Buprenorphine SR (delayed release; 0.20 mg/kg) was administered subcutaneously prior to surgery for prophylactic pain management.

Six full thickness wounds were created along the dorsum of each animal, between the shoulder and ilium, with penetration through the subcutaneous layer to fascia across the entire wound bed. Each wound was approximately 3 cm in diameter (7.1 cm^2^), with the wounds spaced 3 cm apart. Each wound on the left of the dorsum (control) was dressed with a bilayer matrix consisting of cross-linked bovine collagen and a semi-permeable silicone (Integra® Bilayer Matrix Wound Dressing, Integra, Plainsboro, NJ). Each wound on the right side (treatment) was dressed with a fully synthetic matrix consisting of electrospun, nonwoven nanofibers (Restrata^TM^, Acera Surgical, St. Louis, MO). The Restrata matrix is comprised of polygalactin 910 and polydioxanone, both of which are biocompatible with known resorption profiles.

**Figure 1:**
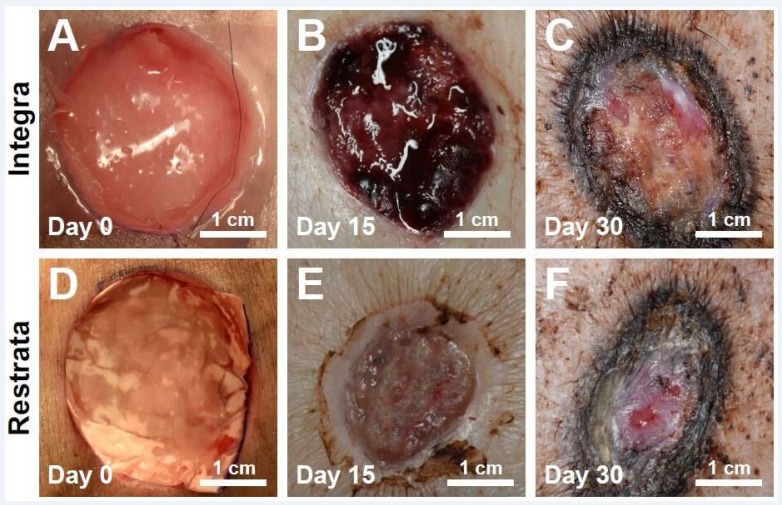
Full thickness cutaneous wounds immediately after application, at Day 15 and Day 30 for Integra Bilayer Wound Matrix (control) (A-C) or Restrata Wound Matrix (treatment) (D-F).

Prior to application, all dressings were soaked in saline, then trimmed to 3.75 cm x 3.75 cm (treatment) and 5.1 cm x 4.2 cm (controls). The trimmed matrices were applied directly to the wounds, ensuring complete contact along the bottom and sides of each wound site (Figure 1A, 1D). The wounds were covered with barrier dressings including sterile gauze, Tegaderm™ (3M, St. Paul, MN) and GranuFoam™ (KCI, San Antonio, TX), which were secured by a tear-resistant mesh stockinet. Barrier wound dressings were changed 2-3 times per week.

Wound observation and measurement

Wounds were photographed 1-2 times per week to grossly monitor the progression of wound healing. Planimetric analysis of wound photographs was performed to assess wound area (Adobe Photoshop CS6). A modified Bates-Jensen scoring system was used to evaluate the inflammation and healing at the wound site by considering five categories: wound edges, exudate quality, exudate quantity, granulation tissue, and epithelialization. Each category was scored on a scale of one to five, with scores of one indicating full wound healing and minimal inflammation and scores of five indicating minimal wound healing and enhanced inflammation. Scores in each category were summed to obtain a total score for each wound and time point, where healing was associated with lower total scores.

Tissue harvesting and histopathological analysis

One animal was euthanized at Day 15 and one at Day 30. The Day 15 time point was chosen as a halfway marker for characterizing healing progression. Wound tissue was harvested immediately after euthanasia for histopathological analysis by excision of the entire wound area, along with an additional 1 cm of surrounding skin. The excised tissue was preserved in 10% formalin, and embedded in paraffin. The wound tissue was sectioned and stained with hematoxylin and eosin (H&E) and analyzed by light microscopy by Alizée Pathology (Thurmont, MD) for evidence of inflammation and wound healing by considering five categories: inflammation, granulation tissue, collagen maturation, vascularization, and epithelialization. Grades were then assigned on an ordinal scale for each of these five categories for each wound section.

Statistical analysis

Student’s t-tests were used for between-groups comparison of wound area over time. Statistical significance was defined as p < 0.05. Averages were calculated for the Bates-Jensen and histopathological analyses then graphically plotted.

## Results

Wound observation and planimetric analysis

Neither material caused adverse reactions at the wound site. However, the animal euthanized on Day 15 exhibited bleeding from its left-side wounds on Day 15. This bleeding was determined to be caused by the animal rubbing against its enclosure and was not an adverse reaction of the wound matrix material. Histopathological analysis (described below) of the wound areas on the left side of this animal demonstrated characteristics indicative of the wound matrices remaining in contact with the wound beds. Therefore, it was determined that any between-group differences in per-protocol outcome measurements were not confounded by the animal's contact with its enclosure.

**Figure 2:**
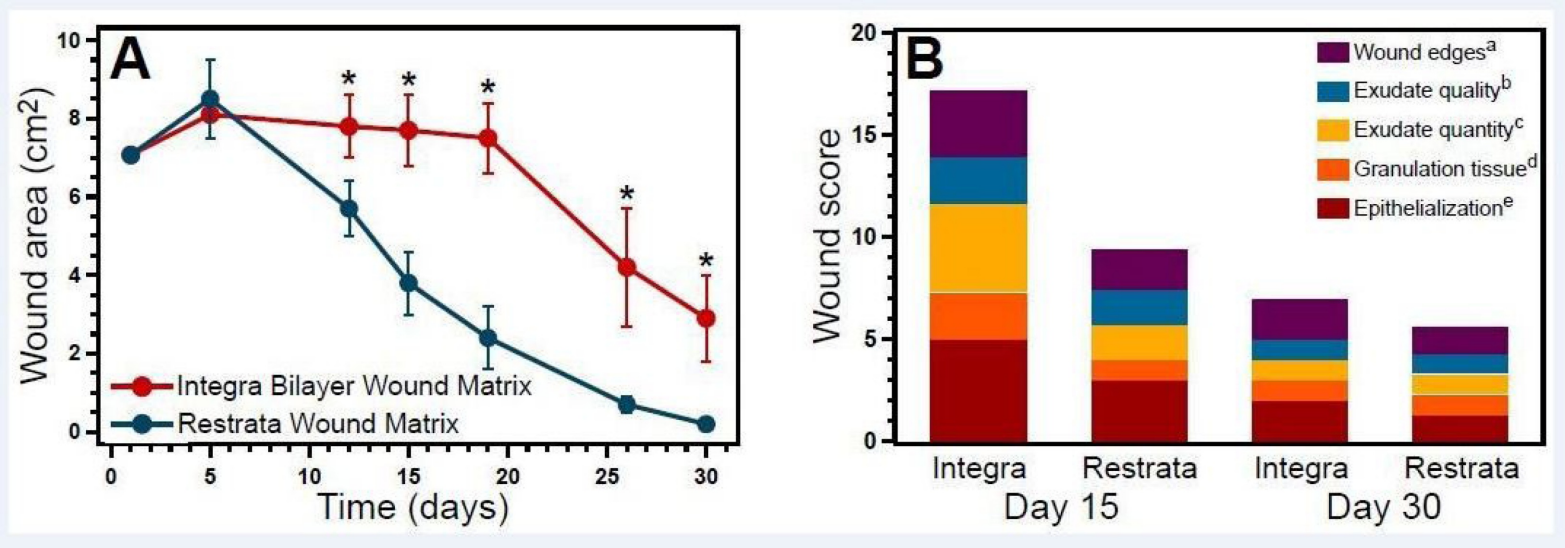
Gross evaluation of inflammation and wound healing. (A) Wound area (average ± SD) as determined by planimetric analysis of wound photographs. *p

Representative photographs of healing progression for all wounds are shown in Figure 1B, 1C and Figure 1E, 1F. Average wound area was 7.1 cm^2^ for both the control and treatment groups at the day of surgery. Days 15 and 30 average wound area was [7.7 cm^2^ ± 0.9]/[3.8 cm^2^ ± 0.8] (p = 0.002) and [2.9 cm^2^ ± 1.1]/[0.2 cm^2^ ± 0.0] (p = 0.01) for controls/treatment. Between-group comparisons of average decrease in wound area over 15 and 30 days were statistically significant (p < 0.05) (Figure 2A). For all time points after Day 5, the average area of wounds with the treatment dressing was smaller than those treated with the control dressing. Two of the three wounds receiving the treatment dressing were fully healed at Day 30 (wound area = 0 cm^2^) (Figure 2A).

At Day 15, wounds treated with the treatment dressing had a Bates-Jensen score that was 46% lower on average than for wounds treated with control dressings (Figure 2B). At Day 30, a 19% difference was found in favor of the treatment dressing (Figure 2B).

Histopathological analysis

**Figure 3:**
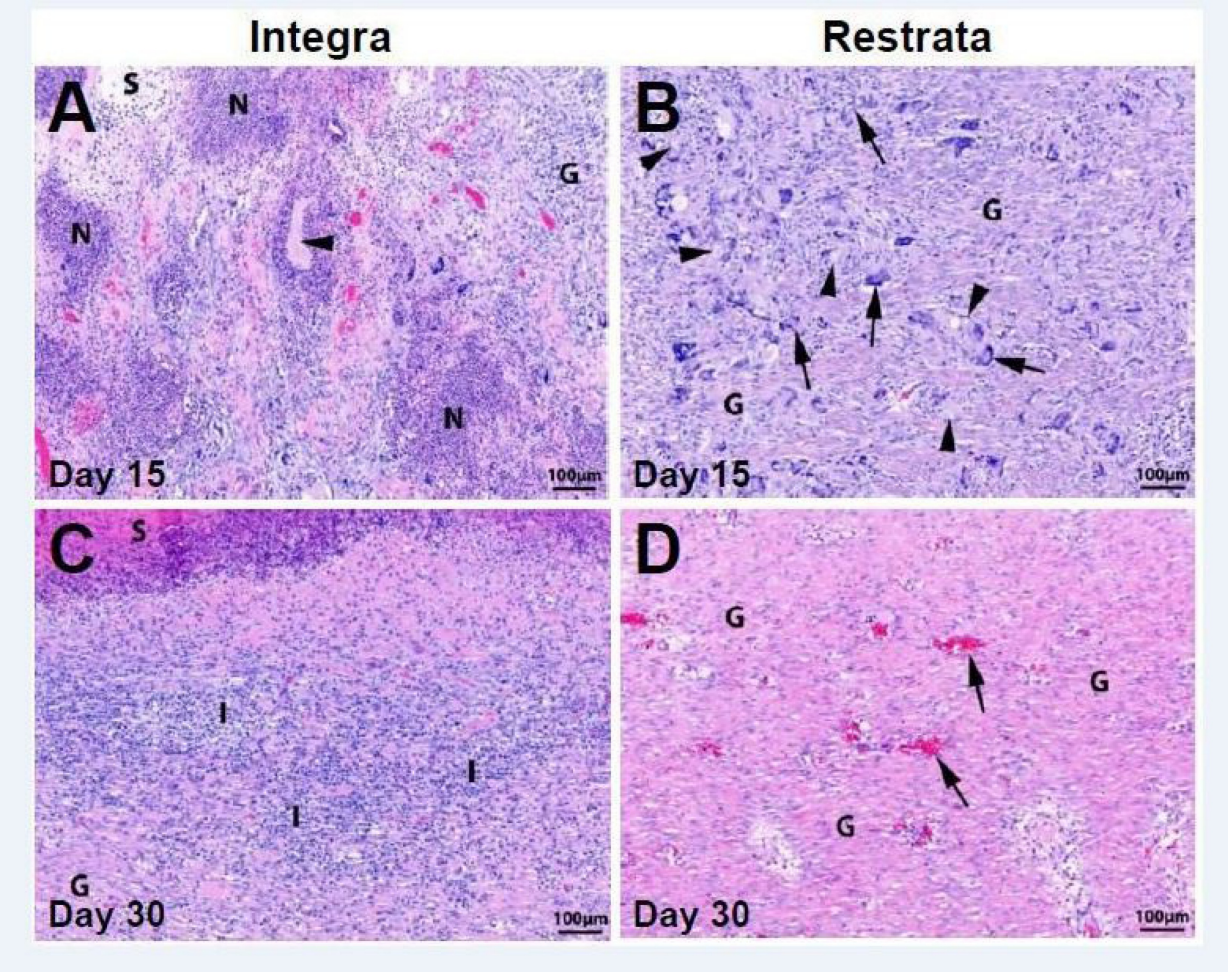
Hematoxylin and eosin (H&E) stained sections from wounds treated with (A) Integra Bilayer Wound Matrix or (B) Restrata Wound Matrix at Day 15. G – granulation tissue, N – neutrophils, S – seroma, Arrowheads – wound matrix material, Arrows – multinucleated giant cells surrounding wound matrix material. H&E stained sections from wounds treated with (C) Integra Bilayer Wound Matrix or (D) Restrata Wound Matrix at Day 30. G – granulation tissue, I – inflammation (infiltrating neutrophils and macrophages), S – serocellular debris. Arrows – blood vessels.

**Figure 4:**
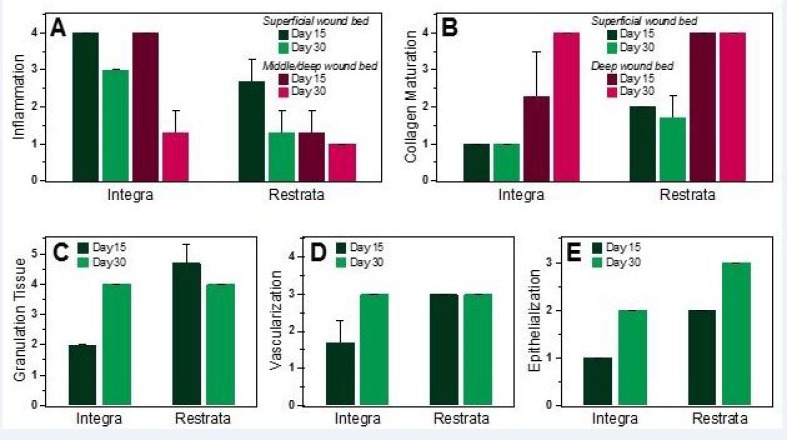
Histopathological scoring (average ± SD) of wounds treated with Integra Bilayer Wound Matrix or Restrata Wound Matrix. Scored for (A) overall inflammation – 0 (absent) to 4 (marked), (B) collagen maturation – 0 (no deposition) to 4 (notable deposition with appearance of native dermal collagen), (C) granulation tissue – 0 (none) to 5 (greater than 100% of wound bed filled with excessive granulation tissue), (D) vascularization – 0 (none) to 4 (numerous blood vessels throughout the entire wound bed), and (E) epithelialization – 0 (none) to 3 (complete coverage by epithelium).

Inflammation of the wound site was assessed by the presence of infiltrating neutrophils, eosinophils, macrophages, and multinucleated giant cells (Figure 3A-3D). Table 1 and Figure 4A-4E display the average scores for each of the five categories used to assess inflammation and wound healing.

**Table 1:** Average histopathological scoring of wounds treated with Integra Bilayer Wound Matrix or Restrata Wound Matrix. (a) Scored from 0 (absent) to 4 (packed). (b) Scored from 0 (absent) to 4 (marked). (c) Scored from 0 (no deposition) to 4 (notable deposition with appearance of native dermal collagen). (d) Scored from 0 (none) to 5 (greater than 100% of wound bed filled with excessive granulation tissue). (e) Scored from 0 (none) to 4 (numerous blood vessels throughout the entire wound bed). (f) Scored from 0 (none) to 3 (complete coverage by epithelium).

	Control		Treatment	
	Day 15	Day 30	Day 15	Day 30
Infiltrating cells^a^				
*Neutrophils*	4.0	2.0	2.3	0.0
*Eosinophils*	2.3	0.0	0.0	0.7
*Macrophages*	4.0	3.0	2.7	1.3
*Multinucleated giant cells*	3.0	0.7	2.7	1.0
Inflammation^b^				
*Superficial wound bed*	4.0	3.0	2.7	1.3
*Middle/deep wound bed*	4.0	1.3	1.3	1.0
Collagen maturation^c^				
*Superficial wound bed*	1.0	1.0	2.0	1.7
*Deep wound bed*	2.3	4.0	4.0	4.0
Granulation tissue^d^	2.0	4.0	4.7	4.0
Vascularization^e^	1.7	3.0	3.0	3.0
Epithelialization^f^	1.0	2.0	2.0	3.0

At Day 15, wounds receiving the treatment dressing exhibited moderate inflammation, with neutrophils, lymphocytes, and macrophages infiltrating the wound, particularly in the superficial wound bed. In control dressing wounds, more significant inflammation was present as evident from the greater infiltration of macrophages, lymphocytes, eosinophils, and multinucleated giant cells. Areas of hemorrhaging, necrosis, and seroma were also evident in wounds treated with the control dressing at Day 15.

Inflammation decreased from Day 15 to Day 30 in wounds treated with either wound matrix material. In treatment dressed wounds there was minimal inflammation remaining at Day 30, where lymphocytes, macrophages, multinucleated giant cells, and eosinophils were found only sparsely throughout the wound. At Day 30, wounds with the control dressing exhibited moderate inflammation localized to the superficial wound bed with infiltrating macrophages, lymphocytes, and neutrophils.

Similar amounts of residual matrix material were found at the wound sites treated with either dressing at Day 15. Treatment dressings were present in the wound tissue as small particles surrounded by multinucleated giant cells. Control dressings were present in the wound tissue as clumps of collagen surrounded by numerous inflammatory cells including neutrophils, macrophages, and multinucleated giant cells. By Day 30 no wound matrix material was evident in the wound tissue for either dressing type, confirming the resorption of both matrix materials over the course of one month.

Granulation tissue was evaluated by the degree to which it filled the wound site. At Day 15, wounds treated with the treatment dressing already exhibited granulation tissue covering the entire wound area, with two wounds containing excess granulation tissue beyond the wound bed. In contrast, the granulation tissue in wounds treated with control dressings only covered 20-50% of the wound area at Day 15. By Day 30, wounds with either dressing type exhibited complete coverage with granulation tissue, without excessive granulation tissue beyond the wound site.

The presence and quality of collagen was also evaluated at the wound site. Wounds with treatment dressings exhibited more mature collagen in the superficial and deep wound beds at Day 15 than in wounds with control dressings. By Day 30, wounds with treatment dressings had a slightly greater amount of mature collagen in the superficial wound bed, but wounds treated with either matrix material exhibited similar amounts of mature collagen in the deep wound bed.

The presence and number of blood vessels in the wound sections were used to characterize wound vascularization. Wounds with treatment dressings exhibited greater vascularization at Day 15 compared to wounds treated with control dressings. At Day 30, wounds treated with either wound matrix material exhibited equal degrees of vascularization, where numerous blood vessels were present in the wound sections.

Epithelialization was evaluated as the amount of epithelium ingrowth from the wound edges. Greater epithelialization of the wound was achieved with treatment dressings compared to controls at both time points. At Day 15, all three wounds receiving treatment dressings had greater than 1 mm ingrowth of epithelium beyond the wound edges, while wounds treated with control dressings all had less than 1 mm ingrowth of epithelium. By Day 30, all three wounds receiving treatment dressings were completely covered by epithelium, whereas all wounds with control dressings had greater than 1 mm ingrowth of epithelium, but were not fully re-epithelialized.

## Discussion

The multifaceted etiology of wound healing has prompted the development and characterization of a broad spectrum of wound matrix technologies. Full synthetics are of particular interest given the need to identify an ideal material that minimizes risks of biologic matrices while promoting complete healing [11]. This study assessed a fully synthetic nanofiber matrix comparing it to a commercially available, ‘gold standard’ xenograft.

We recognize the potential confounding effect on Day 15 results based on the one animal's left side contact with its enclosure. This concern was alleviated by histopathological findings that were consistent with stable, complete contact between control matrices and the wound beds. In addition, the wound area and histopathological findings in the undisturbed animal sacrificed at Day 30 were indicative of the control group's wound healing trajectory identified in Day 15 analysis. Both animals were of the same strain with no variance in how they were treated and maintained up until the time of sacrifice.

While both treatment and control matrices promoted wound healing in full thickness cutaneous wounds, marked differences were seen between groups where wounds treated with the treatment dressings exhibited less inflammation and more complete wound healing. Furthermore, healing was accelerated in wounds with treatment matrices compared to those treated with control matrices.

Wound area decreased over time for wounds treated with either material, supporting the ability of both to heal full thickness cutaneous wounds. However, the average wound area of wounds with treatment matrices was statistically smaller than wounds treated with control matrices at all time points following Day 5. Compared to Day 5 planimetric measurements, treatment group wounds decreased by 98% in average wound area by Day 30, while control group wounds decreased by 64%. By Day 30, two of the three treatment group wounds had a wound area of 0 cm^2^, indicating complete healing. Such extensive wound surface area reduction in the treatment group compares well to trends observed by Wang, et al. when characterizing a novel collagen-peptide bound synthetic scaffold in a full-thickness porcine model [12]. At 28 days, Wang, et al. concluded that wounds treated with the novel bioactive scaffold exhibited a 96.9% reduction in size. While similar outcomes were noted for the two synthetic matrices, consideration must be given to both the bioactive design of the collagen-peptide scaffold and the step-wise synthesis production process required. The fully-synthetic treatment matrix assessed in this study is constructed via electrospinning to achieve a polymer scaffold mimicking the structure of extracellular matrix without the introduction of a separately sourced collagen component. Risks of bio-incompatibility and diminished availability/scalability with the fully-synthetic treatment dressing are therefore mitigated.

Histopathological analysis of wound sections corroborated the evidence of faster and more complete wound healing in the treatment group. Although inflammation decreased for both dressings between Day 15 and Day 30, treatment group wounds exhibited the lowest inflammation at Day 30. The nanoscale architecture and resorbable nature of the treatment material may explain the reduced inflammatory reaction observed over the course of wound healing, as compared to the bovine nature of the control dressing. By permitting rapid cellular infiltration into the nanofiber matrix and presenting structural features on a size scale comparable to native extracellular matrix components the treatment material induces a blunted inflammatory response distinct from both that elicited by biologic xenogenic or human allogenic materials and macro-scale non-resorbable synthetic matrices.

Treatment matrices induced more rapid wound healing as evidenced by the earlier appearance of granulation tissue completely covering the wound area. Treatment wounds contained granulation tissue covering the entire wound surface as early as Day 15, whereas control wounds contained granulation tissue over only 20-50% of the wound area at this time point. Wounds treated with either wound matrix material achieved complete coverage with granulation tissue by Day 30. Furthermore, the pronounced granulation observed in the treatment group coincides with early and pervasive vascularization in 15 days. Sufficient granulation during the early healing period is critical in support of angiogenesis.

Finally, treatment wounds achieved superior healing response in terms of epithelialization, inducing greater ingrowth of epithelium beyond the wound edges at both Day 15 and Day 30, as compared to controls. Notably, by Day 30 all three wounds with the treatment dressing were completely epithelialized, whereas none of the control wounds achieved full re-epithelialization. Given that diminished re-epithelialization can be a concern with synthetic dressings due to a lacking in basement membrane, these trends with the treatment dressing are particularly encouraging.

## Conclusions

Both Restrata Wound Matrix and Integra Bilayer Wound Matrix successfully supported wound healing in full thickness cutaneous wounds. However, Restrata exhibited superior biocompatibility and reduced inflammation, leading to improved wound healing compared to Integra Bilayer Wound Matrix. Restrata accelerated wound healing, inducing faster rates of wound closure with granulation tissue, as well as achieving deposition of mature collagen and vascularization at earlier time points than in wounds treated with Bilayer Wound Matrix. The superior wound healing achieved with Restrata may represent the ideal wound healing material and benefit diverse applications for partial and full thickness wounds, chronic wounds (e.g., ulcers), and severe wounds caused by trauma or surgery.
